# Utilization of cervical cancer screening among migrants and non-migrants in Germany: results from a large-scale population survey

**DOI:** 10.1186/s12889-019-8006-4

**Published:** 2020-01-06

**Authors:** Patrick Brzoska, Tuğba Aksakal, Yüce Yilmaz-Aslan

**Affiliations:** 1 Health Services Research, Faculty of Health, School of Medicine, Witten/Herdecke University, Alfred-Herrhausen-Straße 50, 58448 Witten, Germany; 2 Bielefeld University, School of Public Health, Department of Epidemiology & International Public Health, 33501 Bielefeld, Germany

**Keywords:** Screening, Cervical cancer, Migrants, Germany, Survey, Utilization

## Abstract

**Background:**

Studies from European and non-European countries have shown that migrants utilize cervical cancer screening less often than non-migrants. Findings from Germany are inconsistent. This can be explained by several limitations of existing investigations, comprising residual confounding and data which is restricted to only some regions of the country. Using data from a large-scale and nationwide population survey and applying the Andersen Model of Health Services Use as the theoretical framework, the aim of the present study was to examine the role that different predisposing, enabling and need factors have for the participation of migrant and non-migrant women in cervical cancer screening in Germany.

**Methods:**

We used data from the ‘German Health Update 2014/2015’ survey on *n* = 12,064 women ≥20 years of age. The outcome of interest was the participation in cancer screening (at least once in lifetime vs. no participation). The outcome was compared between the three population groups of non-migrants, migrants from EU countries and migrants from non-EU countries. We employed multivariable logistic regression to examine the role of predisposing, enabling and need factors.

**Results:**

Non-EU and EU migrant women reported a lower utilization of cervical cancer screening (50.1 and 52.7%, respectively) than non-migrant women (57.2%). The differences also remained evident after adjustment for predisposing, enabling and need factors. The respective adjusted odds ratios (OR) for non-EU and EU migrants were OR = 0.67 (95%-CI = 0.55–0.81) and OR = 0.80 (95%-CI = 0.66–0.97), respectively. Differences between migrants and non-migrants were particularly pronounced for younger age groups. Self-rated health was associated with participation in screening only in non-migrants, with a poorer health being indicative of a low participation in cancer screening.

**Conclusions:**

The disparities identified are in line with findings from studies conducted in other countries and are indicative of different obstacles this population group encounters in the health system. Implementing patient-oriented health care through diversity-sensitive health services is necessary to support informed decision-making.

## Background

Cervical cancer is the seventh most frequent type of incident cancer among women in Europe [1]. Unlike other, more prevalent, types of cancer, such as carcinoma of the lung, effective screening by means of the Papanicolaou (Pap) smear test is available, which has shown to reduce cervical cancer incidence by 60 to 90% and cervical cancer mortality by up to 90% [2]. Given its effectiveness, regular Pap smear testing is recommended by the World Health Organization [3], the European Union [4] and different national bodies for women from the age of 20 or 25 years up to the age of 65 years or older.

Non-utilization of cancer screening among women and men is associated with lower socioeconomic status [5–7] as well as with other determinants such as poor knowledge about cancer prevention [8] and poor self-rated health [7]. Disparities are particularly pronounced for migrant communities, which constitute increasingly large proportions in many European countries [9]. Similar to breast cancer screening [10–12], many studies conducted in Europe have shown that migrant women participate in cervical cancer screening less frequently than the respective majority populations [13–17]. Comparable findings were reported from other regions of the world such as the United States [18], Canada [19] and Australia [20]. By means of multivariable analyses, all of these studies also showed that differences between migrant and non-migrant females with respect to the utilization of cervical cancer screening are only partially attributable to the influence of demographic and socioeconomic factors. In addition, qualitative studies have revealed that migrants encounter different types of barriers in the health care system, such as poor language proficiency and needs and expectations not sufficiently accounted for by health care providers, contribute to this differential [6, 21].

In Germany, around one quarter of the population are migrants, comprising individuals who themselves or whose parents immigrated to the country after 1949 [22]. Annual Pap smears are recommended for women who are 20 years of age or older [23]. Same as many other types of prevention measures and health care in general, participation in regular Pap smears is covered by individuals’ social insurance and is therefore free of charge for all women, including migrants with a residence status (refugees and asylum seekers without a residence status are only entitled to a limited set of services [24]). Studies show that migrants in Germany – similar to migrants in other European countries – utilize preventive services less frequently than the majority population [25]. In terms of cancer screening, however, results are inconsistent. For example, while some studies reported higher rates of participation in breast cancer screening [26], others showed no differences [27] or considerably lower utilization rates [28]. Little is known about the uptake of cervical cancer screening. One register-based study in the federal state of North Rhine-Westphalia showed lower odds for regular screening in different groups of migrants as compared to the majority population [27]. Another study, using data from a large health insurance organization, revealed slightly higher odds of participation for migrants as compared to the majority population [29]. Both studies are limited since they only focus on selected regions in Germany. The available information, based on administrative data, was also limited and a comprehensive framework such as the Andersen Model of Health Services Use [30] could not be used to examine disparities potentially resulting in residual confounding. Overall, little is known about which demographic, social, behavioral and health-related factors influence the decision of migrant women to utilize cancer screening and whether these factors differ from those in non-migrant women. By means of data from a large-scale and nationwide population survey and applying the Andersen Model of Health Services Use as the theoretical framework, the aim of the present study was to examine the role these factors have for the participation in cervical cancer screening among migrant and non-migrant women in Germany. Insights gained can contribute to inform the implementation of diversity-sensitive services in Germany and other countries which aim to reduce disparities in access to cancer prevention.

## Methods

### Data

The analysis uses data from the ‘German Health Update 2014/2015’, a cross-sectional survey conducted by the Robert Koch-Institute, a research body of the German Federal Ministry of Health [31], between November 2014 and July 2015. Data was collected by means of a standardized self-administered online or postal questionnaire depending on the preference of the respondents. The two-stage cluster sample comprised a total of 24,016 male and female individuals aged 18 years or older who lived in private households and who were registered in population registers with their principal residence. The survey was implemented as part of the routinely conducted health reporting activities of the Robert Koch-Institute and fulfils all requirements and guidelines of the Federal data protection act. Participation in the survey was voluntary and anonymous and all participants provided informed consent before participation [31]. The survey was approved by the Federal Commissioner for Data Protection and Freedom of Information. Following national guidelines for secondary data analyses, no further ethical approval was necessary for the present analysis [32].

Given that in Germany cervical cancer screening is recommended for women 20 years of age or older, we only included women of that age group in the analysis, resulting in a sample size of *n* = 12,725.

### Study variables

In the survey, women had been asked about the last time they underwent a Pap smear, with the response categories being “within the last 12 months”, “1 to less than 2 years ago”, “2 to less than 3 years ago”, “3 years or more ago” and “never”. For the present study, we applied a conservative assessment of participation and distinguished between women who have participated at least once in their life time and those who have never participated.

We compared the participation in cervical cancer screening between the three population groups of non-migrants, migrants from EU countries and migrants from non-EU countries. In line with the standardization employed in the European Health Interview Survey, respondents were regarded as migrants if they were either born outside of Germany or have a non-German nationality [33].

Based on the Andersen Model of Health Services Use [30], we took different predisposing, enabling and need factors as covariates into account. As predisposing factors, we considered, *sex*, *age* (five-year age groups treated as a continuous measure), *partnership status* (living with a partner vs. not living with a partner) and *socioeconomic status (SES)* (low, middle and high). The SES was a standardized summary measure based on vocational education, occupational status and net equivalent income [34]. As enabling factors, we considered the s*ocial support* (poor, moderate, strong) as measured by the Oslo-3 Social Support Scale [35]*, place of residence* (West Germany, East Germany) and the *type of residential area* (rural, small towns [5000–19,999 residents], medium-sized towns [20,000–99,999 residents] and cities [> 99,999 residents]) [36]. As need factors, we took into account the *self-rated health status* (based on a mean score with responses ranging from 1 [“very good”] to 5 [“very poor”]) and the *presence of chronic diseases* (no, yes). All variables included in the analysis had less than 2% of values missing.

### Analysis

We used chi-square (χ^2^) tests and analysis of variance for purposes of sample description where appropriate. For all tests, the significance level was set to *p* < 0.05. To examine differences in the utilization of cervical cancer screening between the three population groups adjusted for predisposing, enabling and need factors, we used a multivariable logistic regression main effects model reporting odds ratios (OR) and their 95%-confidence intervals (95%-CI) as effects estimates. To examine the moderating effects of these factors, in a subsequent step, we included interaction terms between each of the factors and migration status into the model one-by-one [37]. The evaluation of moderating effects was based on average marginal effects (AME) given that ORs may be biased by unobserved heterogeneity [38]. We conduced all analyses using Stata 15 [39].

## Results

Of the *n* = 12,725 women aged 20 and above, 12,064 provided information on all variables and were included in further analysis. Of these, 4.1% were migrants from EU countries and 4.1% were migrants from non-EU countries. The population groups differed by some of the predisposing, enabling and need factors (Tab. 1). Particularly, non-EU migrants were younger, had a lower socioeconomic status and perceived less often strong social support than non-migrants. Among both groups of migrants, the percentage living with no partner, residing in rural areas as well as in the Eastern part of Germany was considerably lower than among non-migrants. In terms of participation in cervical cancer screening, non-EU and EU migrant women reported a significantly lower utilization (50.1 and 52.7%, respectively) than non-migrant women (57.2%).

**Table 1:** Description of the study sample by migrant status (German Health Update 2014/2015, women age 20 years and above, *n* = 12,064)

	Population group			*p*-value*
	Non-migrants	Migrants from EU countries	Migrants from non-EU countries	
N	11,081	490	493	
Age				< 0.01
20–39 years	3346 (30.2%)	157 (32.0%)	225 (45.6%)	
40–59 years	4501 (40.6%)	198 (40.4%)	180 (36.5%)	
60 + years	3234 (29.2%)	135 (27.6%)	88 (17.8%)	
Partnership status				< 0.01
Partner	6239 (56.3%)	307 (62.7%)	306 (62.1%)	
No partner	4842 (43.7%)	183 (37.3%)	187 (37.9%)	
Socioeconomic status				< 0.01
Low	1579 (14.2%)	74 (15.1%)	105 (21.3%)	
Moderate	6622 (59.8%)	251 (51.2%)	252 (51.1%)	
High	2880 (26.0%)	165 (33.7%)	136 (27.6%)	
Social support				< 0.01
Low	1709 (15.4%)	101 (20.6%)	118 (23.9%)	
Moderate	5858 (52.9%)	256 (52.2%)	269 (54.6%)	
High	3514 (31.7%)	133 (27.1%)	106 (21.5%)	
Region				< 0.01
Western Germany	8118 (73.3%)	436 (89.0%)	439 (89.0%)	
Eastern Germany	2963 (26.7%)	54 (11.0%)	54 (11.0%)	
Type of residential area				< 0.01
Cities	3340 (30.1%)	205 (41.8%)	225 (45.6%)	
Medium-sized towns	3872 (34.9%)	196 (40.0%)	201 (40.8%)	
Small towns	1768 (16.0%)	54 (11.0%)	39 (7.9%)	
Rural	2101 (19.0%)	35 (7.1%)	28 (5.7%)	
Self-rated health status [1 “very good” to 5 “very poor”], mean (SD)	2.2 (0.8)	2.1 (0.8)	2.2 (0.8)	0.04
Presence of chronic diseases				0.07
No	5881 (53.1%)	272 (55.5%)	285 (57.8%)	
Yes	5200 (46.9%)	218 (44.5%)	208 (42.2%)	
Utilization of cervical cancer screening				< 0.01
Yes (at least once in life time)	6342 (57.2%)	258 (52.7%)	247 (50.1%)	
No	4739 (42.8%)	232 (47.3%)	246 (49.9%)	

* *p*-value from chi-square test for categorical variables and analysis of variance for continuous variables

These differences also remained evident after adjustment for the role of predisposing, enabling and need factors. As the main effects logistic model (Tab. 2) shows, non-EU and EU migrant women had 33 and 20%, respectively, lower odds of participation in cancer screening than non-migrant women (OR = 0.67, 95%-CI = 0.55–0.81 and OR = 0.80, 95%-CI = 0.66–0.97, respectively).

**Table 2:** Results of the multivariable logistic regression model with utilization of cervical cancer screening as the dependent variable. Odds ratios (OR) and 95% confidence intervals (95%-CI) (German Health Update 2014/2015, women age 20 years and above, *n* = 12,064; Main effects model. No interaction effects included)

Independent variable	Odds Ratio	95%-CI	*p*-value
Population group (Ref.: Non-migrants)			
Migrants from EU countries	0.80	0.66; 0.97	0.02
Migrants from non-EU countries	0.67	0.55; 0.81	< 0.01
Age	0.86	0.85; 0.87	< 0.01
Partnership status (Ref.: No partner)			
Partner	1.59	1.47;1.73	< 0.01
Socioeconomic status (Ref.: Low)			
Moderate	1.49	1.33; 1.66	< 0.01
High	1.83	1.61;2.09	< 0.01
Social support (Ref.: Low)			
Moderate	1.28	1.15; 1.42	< 0.01
High	1.46	1.30; 1.65	< 0.01
Region (Ref.: Western Germany)			
Easters Germany	1.18	1.07; 1.30	< 0.01
Type of residential area (Ref.: Cities)			
Medium-sized towns	0.95	0.86; 1.04	0.25
Small towns	1.04	0.92; 1.17	0.56
Rural	0.50	0.85; 1.08	0.50
Self-rated health status [1 “very good” to 5 “very poor”]	0.85	0.80; 0.90	< 0.01
Presence of chronic diseases (Ref.: No)			
Yes	1.23	1.13; 1.35	< 0.01

Except for the type of residential area, all of the predisposing, enabling and need factors studied were significantly associated with utilization. A younger age, living together with a partner, having a higher socioeconomic status and higher social support were associated with higher odds of participation in cervical cancer screening. Similarly, women with chronic conditions were more likely to have utilized screening at least once before the survey. Conversely, higher age and worse self-perceived health status were associated with lower odds of having received a Pap smear before the survey.

**Fig. 1:**
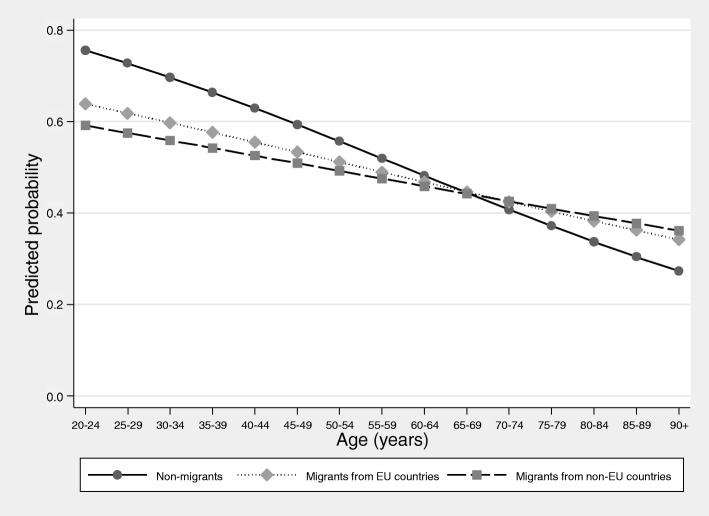
Probability of the utilization of cervical cancer screening by population group and age. Results of the multivariable logistic regression model with utilization of cervical cancer screening as the dependent variable and interaction effects between age and population group. (German Health Update 2014/2015, women age 20 years and above, *n* = 12,064; results from logistic regression model with interaction effects between age and migrant status)

**Fig. 2:**
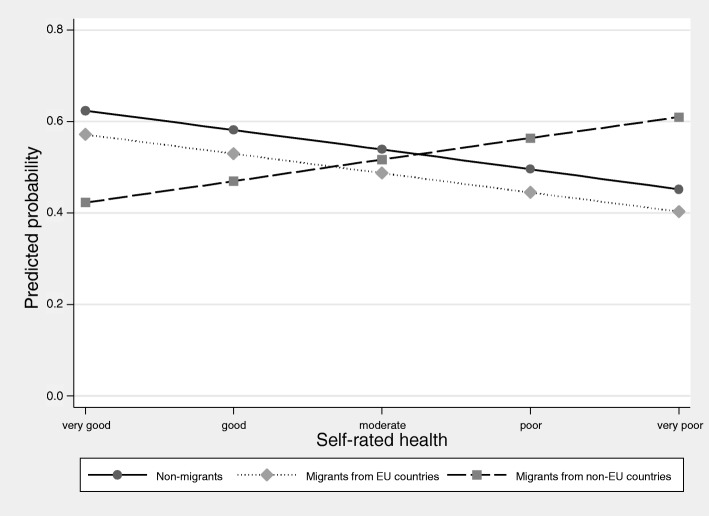
Probability of the utilization of cervical cancer screening by population group and self-rated health status. Results of the multivariable logistic regression model with utilization of cervical cancer screening as the dependent variable and interaction effects between self-rated health and population group. (German Health Update 2014/2015, women age 20 years and above, *n* = 12,064; results from logistic regression model with interaction effects between self-rated health and migrant status)

An investigation of interaction effects revealed that differences between migrants and non-migrants were particularly pronounced for younger age groups which decreased with age. Conversely, this means that age was only a significant determinant of utilization for non-migrants, with older women being at a lower likelihood of participation in cancer screening (Fig. 1). Self-rated health was associated with participation in screening only in non-migrants, with a poorer health being indicative of a low participation in cancer screening (Fig. 2). The role of other predisposing, enabling and need factors did not significantly differ between the three population groups.

## Discussion

In many countries, migrants utilize cancer screening, including cervical cancer screening, less frequently than the majority population [13–17]. Findings from studies in Germany are inconsistent with some reporting disparities in favor of non-migrants [27] while others observed a higher utilization among migrants [29]. Aside from being restricted to some regions of Germany, a major limitation of these studies is a potential bias through residual confounding since only a limited number of influencing factors could be taken into account. Using large and nationwide survey data, the present study examined predisposing, enabling and need factors for the utilization of cervical cancer screening in migrants and non-migrants in Germany.

The study shows that EU- and non-EU migrant women residing in Germany utilize cervical cancer screening less often than non-migrant women. These differences cannot be explained by a different distribution of predisposing, enabling and need factors between the population groups as a multivariable analysis revealed. The findings are in line with studies from other countries [13–17]. They also suggest that additional factors need to be considered when addressing disparities in the utilization of cervical cancer screening among migrants. Similar to disparities in the utilization of other health services, the lower average level of utilization among migrant women is likely indicative of different obstacles this population group encounters in the health system. These barriers include a limited German language proficiency, lack of information and low health literacy [40]. Furthermore, because of discrimination, lack of awareness and/or insufficient financial resources, health care providers often do not adequately meet the (cultural) expectations migrants have towards health care. These may, for example, comprise religious and cultural taboos or disadvantageous beliefs about illness and treatment [6, 21, 25, 41].

Aside from differences between migrants and non-migrants, the study also identified different predisposing, enabling and need factors relevant for the utilization of cervical cancer screening among the population in Germany. The findings are in line with research from other countries [42–44]. In our study, women living in the Eastern part of Germany had a higher likelihood of cervical cancer screening use. This corresponds to findings from studies based on routine data [45] and can be explained by more thorough screening policies in the former Democratic Republic of Germany before reunification [46].

The study showed that most of the predisposing, enabling and need factors did not significantly differ between migrant and non-migrant women. Age was identified as a moderating factor with disparities between migrant and non-migrant women decreasing with age. Age has also been identified as a moderator for disparities between migrants and non-migrants with respect to the utilization of other health services such as regular dental check-ups [47]. Because no information on the length of stay was available in the data, it remains unclear whether this finding is attributable to acculturation and increased knowledge of the German health care system among those with a longer length of stay [48]. Aside from age, the effect of self-rated health also differed between migrants and non-migrants. A significant association between self-rated health and utilization of cervical cancer screening could only be observed for non-migrants. These differences could be explained by a possible attenuation of the association through particular illness beliefs such as fatalism, which have been shown to affect the uptake of cancer screening and to be associated with self-rated health [49, 50].

Strengths of the present study are the large and nationwide sample as well as high quality of data collection. To the best of our knowledge, it is also the first study applying the Andersen Model of Health Services Use as a comprehensive theoretical framework to study disparities in the utilization of cancer screening among the migrant population in Germany. Some limitations inherent to the data need to be considered as well. First, the study was conducted in German language only. It is therefore likely that migrants with limited German-language proficiency are underrepresented. Since poor German-language proficiency can be a significant barrier with respect to the utilization of health services [25, 51], it can be assumed that our study underestimates the disparities in the utilization of cervical cancer screening among migrants. Aside from language proficiency, we were also not able to take into account heterogeneity with respect to religion, culture, ethnicity, acculturation and length of stay, which previous research has identified to also influence participation in cervical cancer screening [14, 15, 52]. In order to devise more targeted patient-oriented services, future studies need to examine the role of these factors for migrants in Germany. Second, all information, including data on the utilization of cancer screening, were based no self-reports. Evidence on the validity of self-reported utilization of cancer screening is inconclusive [53, 54]. With respect to statutory health checks in Germany, studies have shown that self-reported information collected in the German Health Update survey corresponds to administrative data and can be considered valid [55]. Similarly, the self-reported information on demographic and socioeconomic factors can be considered valid given that the distribution of these factors in the sample resembles that of the total population in Germany in the year the data was collected [56].

## Conclusion

This study was the first to investigate disparities in the uptake of cervical cancer screening among migrant and non-migrant women in Germany using nationwide data.

The lower utilization of migrant as compared to non-migrant women can probably be explained by barriers migrants encounter in health care indicating that the health system is not sufficiently sensitive to the needs and expectations of this population group. Implementing patient-oriented health care through diversity-sensitive health services is necessary to supported informed decision making. This does not only include information taking into account the oftentimes limited health literacy of this population group, but also comprises information and services which consider their cultural needs and expectations.

## Data Availability

Data used in the present study can be obtained from the Robert Koch Institute, Germany (see https://www.rki.de/DE/Content/Forsch/FDZ/informationen_antrag/info_antrag_node.html).

## Abbreviations

95%-CI: 95%-confidence interval
AME: Average marginal effects
EU: European Union
OR: Odds ratio
Pap: Papanicolaou
SES: socioeconomic status
